# The functional modulation of epigenetic regulators by alternative splicing

**DOI:** 10.1186/1471-2164-8-252

**Published:** 2007-07-25

**Authors:** Sergio Lois, Noemí Blanco, Marian Martínez-Balbás, Xavier de la Cruz

**Affiliations:** 1 Instituto de Biología Molecular de Barcelona. CID. Consejo Superior de Investigaciones Científicas (CSIC); 08028 Barcelona, Spain; 2 Institut de Recerca Biomèdica-PCB; 08028 Barcelona, Spain; 3 Institució Catalana de Recerca i Estudis Avançats (ICREA); Barcelona, Spain

## Abstract

**Background:**

Epigenetic regulators (histone acetyltransferases, methyltransferases, chromatin-remodelling enzymes, etc) play a fundamental role in the control of gene expression by modifying the local state of chromatin. However, due to their recent discovery, little is yet known about their own regulation. This paper addresses this point, focusing on alternative splicing regulation, a mechanism already known to play an important role in other protein families, e.g. transcription factors, membrane receptors, etc.

**Results:**

To this end, we compiled the data available on the presence/absence of alternative splicing for a set of 160 different epigenetic regulators, taking advantage of the relatively large amount of unexplored data on alternative splicing available in public databases. We found that 49 % (70 % in human) of these genes express more than one transcript. We then studied their alternative splicing patterns, focusing on those changes affecting the enzyme's domain composition. In general, we found that these sequence changes correspond to different mechanisms, either repressing the enzyme's function (e.g. by creating dominant-negative inhibitors of the functional isoform) or creating isoforms with new functions.

**Conclusion:**

We conclude that alternative splicing of epigenetic regulators can be an important tool for the function modulation of these enzymes. Considering that the latter control the transcriptional state of large sets of genes, we propose that epigenetic regulation of gene expression is itself strongly regulated by alternative splicing.

## Background

Epigenetic regulation of gene expression constitutes a fundamental mechanism by which a series of chromatin modifications allow the normal functioning of the cell under different conditions [1-3]. In particular, these modifications control the repressive effect of chromatin, which limits the access of regulatory proteins to DNA, thus posing serious restraints to biological processes like replication, transcription, etc [4]. In agreement with this, an increasingly large amount of experimental data shows the relevance of chromatin modifications in development [5], disease [6], etc. For example, recent studies indicate that histone modifications are involved in paternal X chromosome inactivation [7,8]. Work from Roopra and colleagues [9] shows that histone methylation regulates the tissue-dependent silencing of neuronal genes. Also, expression of Hox transcription factors is directly related to the presence of histone marks [10].

Chromatin modifications are produced by a series of chromatin-modifying enzymes (epigenetic regulators) that act on chromatin by either introducing histone modifications or by inducing ATP-dependent nucleosome remodelling. Histone modifications usually take place at histone tails and can introduce a wide variety of covalent marks including acetylation, methylation, phosphorylation, etc [2]. These marks provide a simple way to access nucleosomal DNA and normally have different functional consequences [2,11-14]. A synthetic view of the biological role of histone modifications is provided by the histone code hypothesis [1]. According to this hypothesis, the regulatory state of a gene is a function of these modifications and their combinations. Apart from histone-modifying enzymes, enzymes that utilise ATP to modify the nucleosomal structure, altering histone-DNA interactions [15], also give access to nucleosomal DNA. Interestingly, both mechanisms are coordinated and cooperate to finally give access to nucleosomal DNA. For example, it has been recently shown that the SWI/SNF complex is retained to the chromatin only if SAGA or NuA4 acetylate it [16].

As with transcription factors [17,18], the functional activity of chromatin-modifying enzymes must be regulated in order to produce gene expression patterns that are coherent with high-level biological processes, like development or tissue differentiation. However, little is yet known about how this regulation occurs, due to the recent discovery of these enzymes [2,3,19]. Among the possible regulation levels [18], like transcription, translation or mRNA splicing, in this work we have focused on the study of the latter. We have chosen alternative splicing for four different reasons. First, because recent data [20-23] strongly suggest that alternative splicing can introduce functionally relevant changes in chromatin-modifying enzymes. Second, because alternative splicing is already known to play an important role in gene expression regulation by modulating the functional properties of transcription factors [17,18], for example, alternative splicing can change the DNA-binding properties of transcription factors [24]; introduce or eliminate activating domains [25], increase the *in vivo *stability of a given isoform [26], etc. Third, because of the availability, in public databases, of a large amount of unexplored information on alternative splicing patterns of chromatin-modifying enzymes is available in public databases. And fourth, because the functional and regulatory impact of the most frequent alternative splicing events -in particular long sequence insertions/deletions- is relatively easier to infer, particularly if it affects known protein domains [17].

**Figure 1:**
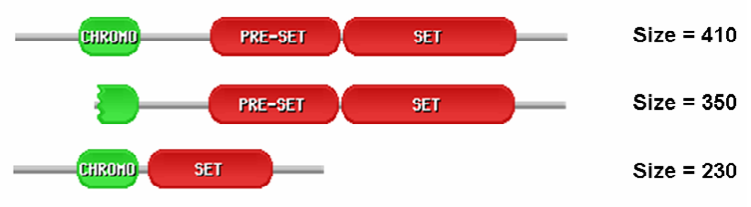
**Alternative splicing pattern of human histone methyltransferase SUV39H2**. Representation of the domain structure of three isoforms of SUV39H2, together with their sizes. Shown in red are the two domains, PRE-SET and SET that constitute the catalytic unit of the enzyme. The interaction domain, chromodomain, is shown in green. This domain is seriously damaged in the second isoform, and is unlikely to play any targeting role. The catalytic unit, on the contrary, remains intact in this second isoform, but is clearly damaged in the third isoform, with 28 % of the SET domain and the whole PRE-SET domain missing.

In our work we have studied (i) whether, and to which extent, epigenetic regulators (ATP-dependent remodelling enzymes, histone acetyltransferases, deacetylases, methyltransferases, etc) have alternative splicing, and (ii) the impact of alternative splicing on the domain structure of these enzymes, with special focus on catalytic and interaction domains, which are known to play a key role [2,3,27,28]. We obtained the alternative splicing data from databases with very different curation protocols, going from literature surveys, like SwissProt [29], to that of highly automated methods based on sequence processing and EST data, like ENSEMBL [30]. Our results show that a substantial percentage of epigenetic regulators, 49 % (70 % for human genes), have alternative splicing. In addition, in more than 59 % of these cases alternative splicing changes affect either the catalytic or the interaction domain (Figure 1), suggesting the existence of functional regulatory effects comparable to those found in transcription factors [17].

## Results and discussion

A set of 160 genes, from different species, of chromatin-modifying enzymes was considered in this work. These enzymes cover the following activities: ATP-dependent chromatin remodelling, histone acetylation, deacetylation, methylation, demethylation, phosphorylation, ubiquitination, and sumoylation. We find (Table 1) that 49 % of the genes show alternative splicing, with an average number of 2.8 isoforms per gene. In humans, this number goes up to 70 % (with 2.8 isoforms per gene), a value close to one of the largest estimates obtained for human, *e.g*. 74 % [31]. This result points to a significant role of alternative splicing in the modulation of the functional properties of chromatin-modifying enzymes.

**Table 1:** Summary of the data utilised in this work

	Number of genes	Number of genes with AS	Number of genes with AS involving protein domains
**All species**	160	78	46
*Homo sapiens*	71	50	32
*Mus musculus*	31	21	10

To characterise the functional variability introduced by alternative splicing in chromatin-modifying enzymes, we compared the different isoforms of the same gene at the protein sequence level, using the longest isoform as a reference. We focused our study on the changes affecting protein domains of known function, because they can be reliably interpreted in terms of biochemical/biological function [17]. For example, it has been experimentally shown that domain changes between isoforms can be associated to isoforms with [17,32]: a dominant-negative role, different binding affinities or new interaction partners, modified enzymatic activity, etc.

In our case, we observe that 60 % (64 % for human) of the genes with alternative splicing have isoforms with at least one missing, or significantly affected, domain (Table 1). These cases can be grouped according to the functional role of the domain: (i) changes in the catalytic domains; (ii) changes in the protein interaction domains; and (iii) drastic sequence reductions. There are only four exceptions to this broad classification, corresponding to the small, single-domain, human proteins: ubiquitin-conjugating enzyme E2A (UBE2A, 154 aas), casein kinase 2, alpha 1 polypeptide (CKII, 391 aas), NAD-dependent deacetylase sirtuin-2 (SirT2, 389 aas) and aurora kinase B (AURKB, 344 aas) for which interaction and catalytic domains coincide. In these cases, alternative splicing modifications will affect both functions.

We discuss below the three above-mentioned scenarios.

### (i) Changes in the catalytic domains

**Figure 2:**
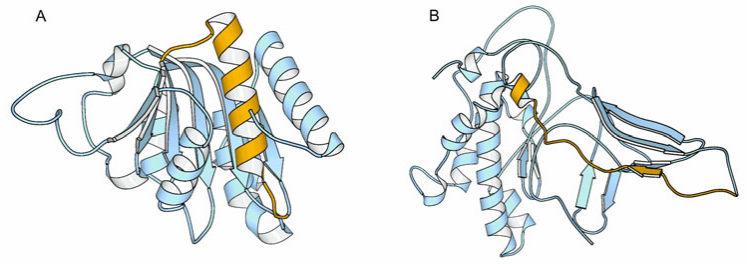
**Impact of alternative splicing in catalytic domains**. In all cases the part of the protein affected by alternative splicing is shown in yellow, while the remaining of the protein is shown in blue. (A) Domain DEXHC of human chromatin remodelling SMARCA1. Alternative splicing results in the loss of a α-helix. (B) Domain PI3_PI4_KINASE of kinase PRKDC. Alternative splicing results in the loss of a sequence stretch that has very distant ends. The figures were obtained using the MOLSCRIPT software [65].

In the human, we find several genes with isoforms that have the catalytic domain either missing or affected (Table 2). In a short isoform of the histone methyltransferase SUV39H2 (Figure 1), the catalytic unit is seriously damaged by the loss of the whole PRESET domain, and about 30 % of the SET domain. The situation seems different for chromatin remodelling SMARCA1's and kinase PRKDC's short isoforms, which only lack 11 % and 8 % of their respective catalytic domains (Table 2). However, visual inspection of the catalytic domains' structures shows that the changes are far from being structurally neutral. The deletion affecting the helicase domain DEXHC of the chromatin-remodelling enzyme SMARCA1 involves an alpha helix linking two of the most extreme strands of the central beta sheet (Figure 2A). The deletion affecting the catalytic PI3_PI4_KINASE domain of the kinase PRKDC affects a beta sheet, eliminating one strand and altering the inter-strand connectivity (Figure 2B). In both cases, the changes will produce either structural strain, or significant rearrangements, likely to result in function loss/modification. Indeed, recent experimental data for kinase PRKDC [23] show that the protein kinase activity of the short isoform of this enzyme is lost.

**Table 2:** Cases for which alternative splicing sequence changes mainly affect catalytic domains

Gene name	Species	Reference Isoform Size	Alternative Isoform Size	Domains affected
SUV39H2	H.s.	410	230	PRESET, SET
SMARCA1 (SNF2L)	H.s.	1054	1033	DEXHC
PRKDC (DNA-PK)	H.s.	4127	4097	PI3_PI4 KINASE
RPS6KA5 (MSK1)	H.s.	802	549	PKINASE
EZH2	H.s.	751	376	SET
EHMT2 (G9a)	H.s.	1210	202	ANK, PRESET, SET*
CARM1 (PRMT4)	H.s.	608	412	SKB1
SETDB1	H.s.	1290	397	MBD, PRESET, SET, TUDOR
EHMT1	H.s.	1267	1153	SET
FBXL11 (JHDM1A)	H.s.	1162	856	JMJC
AOF2 (LSD1)	H.s.	876	852	AMINO_OXIDASE
GSG2 (HASPIN)	H.s.	798	314	PKINASE
PRDM2 (RIZ1)	H.s.	1718	1481	SET
Setdb1	M.m.	1308	488	MBD, PRESET, SET
Htatip	M.m	546	492	MOZ_SAS
Fbxl10 (Jhdm1b)	M.m.	1309	776	JMJC
Fbxl10 (Jhdm1b)	M.m	1309	656	JMJC
Jmjd1b (Jhdm2b)	M.m	1562	1124	JMJC
fbxl10 (Jhdm1b)	X.l.	1259	738	JMJC
mez2	Z.m.	894	624	SET

Inactivation of the enzyme's catalytic function by alternative splicing is also found in one of maize methyltransferase mez2's isoforms that has completely lost its SET domain (Table 2).

Two cases deserve additional comment. CARM1 (coactivator-associated arginine methyltransferase 1) has an alternative splice isoform, the catalytic domain of which, SKB1, is clearly damaged (48 % of the domain is lost). We have classed CARM1 within this section, even though an interaction domain has not yet been identified, because the full-length isoform is big enough (608 aas) to have both an interaction domain and a catalytic domain. The second case is that of RPS6KA5 (ribosomal protein S6 kinase, 90 kDa, polypeptide 5) which has two catalytic domains, but no interaction domain. In this case, lack of one of the catalytic domains may result in either an inactive or a less active protein. This situation would be equivalent to an amount regulatory mechanism similar to that described for other enzymes.

In general, alternative splicing isoforms with a missing catalytic domain may behave as dominant-negative regulators of the fully functional isoform, a well-known situation in the case of transcription factors [17,33]. This may be the case in chromatin-modifying enzymes. Indeed, a recently described PRKDC isoform with no protein kinase domain has no catalytic activity and shows slight inhibitory activity of the full-length isoform [23]. However, the situation may be more complex, as for example the short PRKDC isoform described here is able to participate in some DNA repair processes, despite having no kinase activity [23]. Thus we cannot rule out the possibility that, in some cases, isoforms lacking the catalytic unit may have functional roles other than being dominant-negative regulators.

### (ii) Changes in the protein interaction domains

As for the previous case, the effect of alternative splicing can range from partial deletion to complete domain loss (Table 3). In the human, we find the latter in several genes, for example GCN5L2, MYST1 and MORF4L1. The first of them expresses two isoforms lacking the PCAF_N domain, which is involved in the interaction between the histone acetyltransferase GCN5L2 and CBP. For histone acetyltransferase MYST1, the chromodomain is lost together with a substantial part of the protein, but the catalytic domain is left intact. The case of the histone acetyltransferase MORF4L1 is somewhat surprising, as it is the short isoform that shows the chromodomain, after deletion of a sequence stretch that is in the middle of the domain's sequence in the long isoform [20].

**Table 3:** Cases for which alternative splicing sequence changes mainly affect interaction domains

Gene name	Species	Reference Isoform Size	Alternative Isoform Size	Domains affected
SUV39H2	H.s.	410	350	CHROMO
GCN5L2	H.s.	837	476	PCAF_N
GCN5L2	H.s.	837	427	PCAF_N
MYST-1	H.s.	467	300	CHROMO
SMARCA2 (BRM)	H.s.	1590	1572	BROMO
MLL	H.s.	3969	3931	PHD
MORF4L1	H.s.	362	333	CHROMO
MORF4L1	H.s.	362	323	CHROMO
FBXL10 (JHDM1B)	H.s.	1336	1326	LRR_RI
FBXL10 (JHDM1B)	H.s.	1336	1306	LRR_RI
JMJD2B (JHDM3B)	H.s.	1096	448	PHD, TUDOR
MLL2	H.s.	5265	4957	RING, PHD
MLL3	H.s.	4911	4029	PHD
NSD1	H.s.	2696	2593	PWWP
RNF40	H.s.	1001	838	RING, ZF_C3HC4
Morf4l1	M.m.	362	323	CHROMO
Htatip	M.m.	546	302	CHROMO
Fbxl11 (Jhdm1a)	M.m.	1161	494	ZF_CXXC
Fbxl11 (Jhdm1a)	M.m.	1161	338	ZF_CXXC
Jmjd2a (Jhdm3a)	M.m.	1064	1033	PHD, TUDOR
Jmjd2b (Jhdm3b)	M.m.	1086	1021	TUDOR
cbp-1	C.e.	2056	2045	ZNF_TAZ

**Figure 3:**
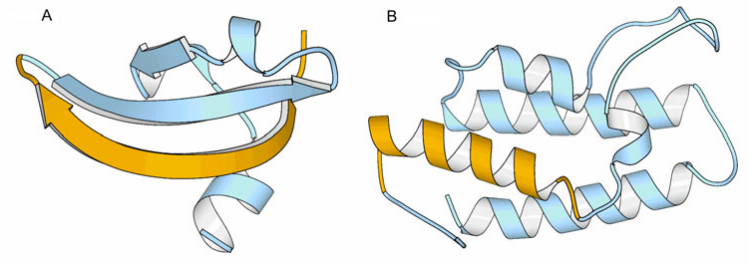
**Impact of alternative splicing in interaction domains**. In all cases the part of the protein affected by alternative splicing is shown in yellow, while the remaining of the protein is shown in blue. (A) Chromodomain of human histone methyltransferase SUV39H2. One of the main strands of the β-sheet is missing in one of the alternative splice isoforms. (B) Bromodomain of human chromatin remodelling SMARCA2. One of the four helices of the helix bundle is lost in the alternative splice isoforms. The figures were obtained using the MOLSCRIPT software [65].

In other cases the impact caused by alternative splicing changes is such that, from a functional point of view, it is essentially equivalent to a domain loss. In general, a simple measure, like size, is usually enough to understand the damaging nature of the change. This is the case of human histone methyltransferase SUV39H2 that has an isoform with only 68 % of its chromodomain (Figure 1). The deleterious effect of this deletion on protein function is supported by visual inspection of the corresponding domain structure that points to a disruption of important secondary structure elements (Figure 3A). Interestingly, even small changes are likely to inactivate the domain's function. For example, chromatin remodelling SMARCA2's bromodomain only looses 14 % of its residues, but analysis of the three-dimensional structure shows that a relevant alpha helix from the helix bundle structure is lost, pointing to a disruption of such a small structure (Figure 3B).

Lack of a whole interaction domain is also found in other species, for example in the short isoform of the mouse histone acetyltransferase Htatip (Tip60), which has a missing chromodomain (Table 3). It has to be noted that in this case a significant part of the protein is also missing (the short isoform is about half the size of the long isoform). Thus, while the catalytic domain, MOZ_SAS, is preserved, it may happen that some unknown domains are also lost. Interestingly, the case of the human histone acetyltransferase MORF4L1 also appears in mouse.

In all these cases the *a priori *functional meaning of the loss of protein interaction domains is similar and would correspond to a down-regulation of the enzyme's activity. The underlying molecular mechanisms will vary depending on the nature of the interaction lost with the missing domain. If this interaction is required for the formation of a complex between the enzyme and its partners, necessary for the catalysis, down-regulation will result from the formation of inactive complexes. This is probably the case of the short isoform of histone acetyltransferase GCN5L2.

If the missing domain is responsible for substrate targeting, e.g. a chromodomain or a bromodomain, down-regulation will be a consequence of the enzyme being unable to reach its substrate. However, in this case another option is also possible, as the enzyme could be recruited to its reaction site after binding one of its complex's partners. The resulting effect on the regulation of gene expression may be substantially different in this case, as modification of the histone tail will take place. However, lack of the chromatin-binding domain will eliminate the positive feedback in chromatin signalling. The latter is mediated by specific interactions between the modified histone tails and the corresponding enzymes and leads to self-perpetuation of activating marks on chromatin. This effect has been recently proposed for enzymes carrying the bromodomain [16,34].

**Figure 4:**
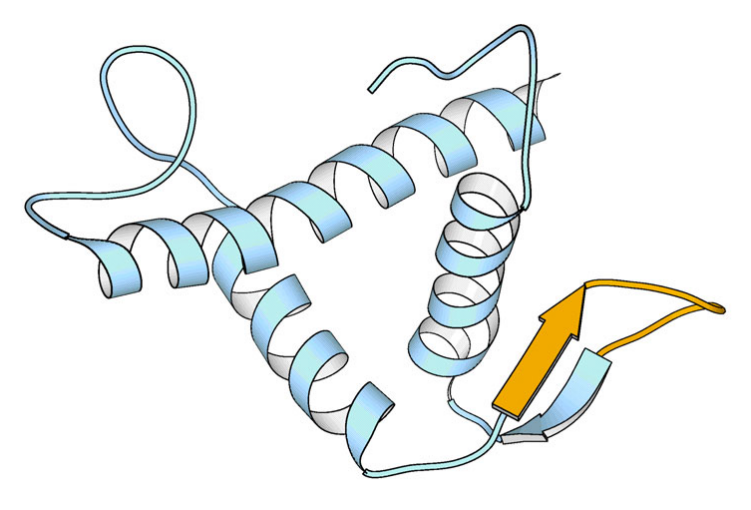
**Impact of alternative splicing in the ZNFTAZ domain of *C.elegans*'s histone acetyltransferase cbp-1**. A small strand (yellow) is lost in one of the alternative splice isoform. Only small changes can be expected from this deletion. The figure was obtained using the MOLSCRIPT software [65].

Lastly, we also find instances where alternative splicing is likely to result in small modulatory changes. For example, in histone methyltransferase MLL only one of the three PHD domains is affected by alternative splicing. The small size of the change, 11 % of the domain, and the fact that the other two PHD domains remain intact, points to a modulation of the enzyme's binding properties rather than to a complete inactivation. For *C.elegans*'s histone acetyltransferase cbp-1, the situation is similar as only one of the two copies of the protein interaction domain ZNF_TAZ is affected, by a small change that happens at a relatively neutral location (Figure 4).

### (iii) Drastic sequence changes

**Figure 5:**
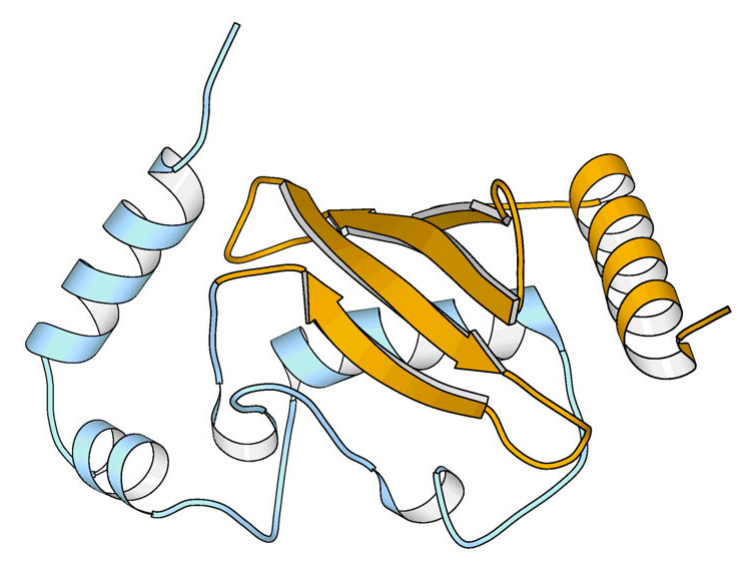
**Alternative splicing of human ubiquitin-conjugating enzyme E2 A UBE2A**. The part of the protein affected by alternative splicing is shown in yellow, and the remaining in blue. One can see that a α-helix and a whole β-sheet are lost in one of the isoforms, with a potentially very disruptive effect. The figure was obtained using the MOLSCRIPT software [65].

Generation of inactive isoforms constitutes a simple and powerful mechanism to regulate the amount of functional protein present in the cell [35-37]. Usually, inactive isoforms are short versions of the fully active protein in which most functional domains are missing [36]. For several genes we find isoforms that fit this description and thus could be inactive isoforms (Table 4). In all of them the size reduction relative to the active protein is dramatic, between 35 % and 95 %, and most of the functional domains are lost or seriously damaged. For example, in the case of the human kinase ATM, the functional protein is 3056 residues long, whilst there is a short isoform associated to this gene with only 138 residues (Table 4). Catalysis-associated domains like FAT, FATC and PI3_PI4_KINASE, are missing from the short isoform, together with most of the non-annotated parts of the sequence. It is improbable that such isoform may have any functional role itself and is thus likely to be the result of the above-mentioned regulatory process. We observe a similar situation for ubiquitin-conjugating enzyme E2 A (UBE2A), which has two isoforms lacking 47 % and 22 % of the UBCC domain. The damaging effect of the missing sequence is supported by visual inspection of the corresponding domain structures (Figure 5).

**Table 4:** Cases for which alternative splicing sequence changes result in drastically affected isoforms

Gene name	Species	Reference Isoform Size	Alternative Isoform Size	Domains affected
SETDB1	H.s.	1290	249	MBD, PRESET, SET, TUDOR
SETDB1	H.s.	1290	151	MBD, PRESET, SET, TUDOR
SMARCA2 (BRM)	H.s.	1590	278	HSA, BRK, DEXHC, HELICASE_C, BROMO
SMARCA2 (BRM)	H.s.	1590	254	HSA, BRK, DEXHC, HELICASE_C, BROMO
SMARCA2 (BRM)	H.s.	1590	236	HSA, BRK, DEXHC, HELICASE_C, BROMO
SMARCA2 (BRM)	H.s.	1590	119	HSA, BRK, DEXHC, HELICASE_C, BROMO
SMARCA4 (BRG1)	H.s.	1679	628	BRK, BROMO, DEXHC, HSA
SUV39H1	H.s.	412	409	CHROMO, PRESET, SET
MLL	H.s.	3969	511	BROMO, FYRC, FYRN, PHD, SET, ZF-CXXC
ATM	H.s.	3056	138	FAT, FATC, PI3_PI4 KINASE
MORF4L1	H.s.	362	235	MRG
EHMT1	H.s.	1267	825	ANK, PRESET, SET
WBP7 (MLL4)	H.s.	2715	582	ZF_CXXC, PHD, FYRC, FYRN, SET
Setdb1	M.m.	1308	500	MBD, PRESET, TUDOR
Stk4	M.m.	487	126	PKINASE
Htatip	M.m.	546	302	CHROMO
Suv39h2	M.m.	477	257	CHROMO, PRESET, SET
Fbxl10 (Jhdm1b)	M.m.	1309	114	JMJC, ZF_CXXC
Su(var)3–9	D.m.	635	475	CHROMO, PRESET, SET
mez2	Z.m.	894	341	SET

In the "Gene name" column we list the standard names of the proteins, although in some cases we also provide alternative names that are frequently used in the literature. In the "Species" column H.s., M.m., D.m., C.e., O.s., X.l. and Z.m. mean *Homo sapiens*, *Mus musculus*, *Drosophila melanogaster*, *Caenorhabditis elegans*, *Oryza sativa, Xenopus laevis *and *Zea mays*, respectively. The sizes of the different isoforms are given in amino acid number. *In this case, although the ANK protein interaction domain is lost, the NFSP transcription factor binding domain is retained.

It has to be noted, however, that short isoforms may not always be the consequence of a regulatory process aiming at reducing the amount of functional protein. In some genes, for example in the case ankyrin-3 [38], they have a specific functional role. This could also be the case for some of the transcripts mentioned in this section.

Furthermore, we cannot completely discard the possibility that some of these cases correspond to database annotation errors.

## Conclusion

A common effect of alternative splicing is to produce isoforms lacking a given functional domain, pointing to an inhibitory role of the fully functional isoforms [17,36,39]. This correspondence between alternative splicing and protein function changes is a consequence of the modular structure of protein function, having been experimentally demonstrated in different instances [17]. Here we show that epigenetic regulators are no exception and that their alternative splicing patterns usually involve loss of the catalytic or the binding domain, resulting in short isoforms that could easily play the above-mentioned inhibitory role. They can also be the consequence of alternative splicing-based mechanisms for the regulation of product amount.

**Figure 6:**
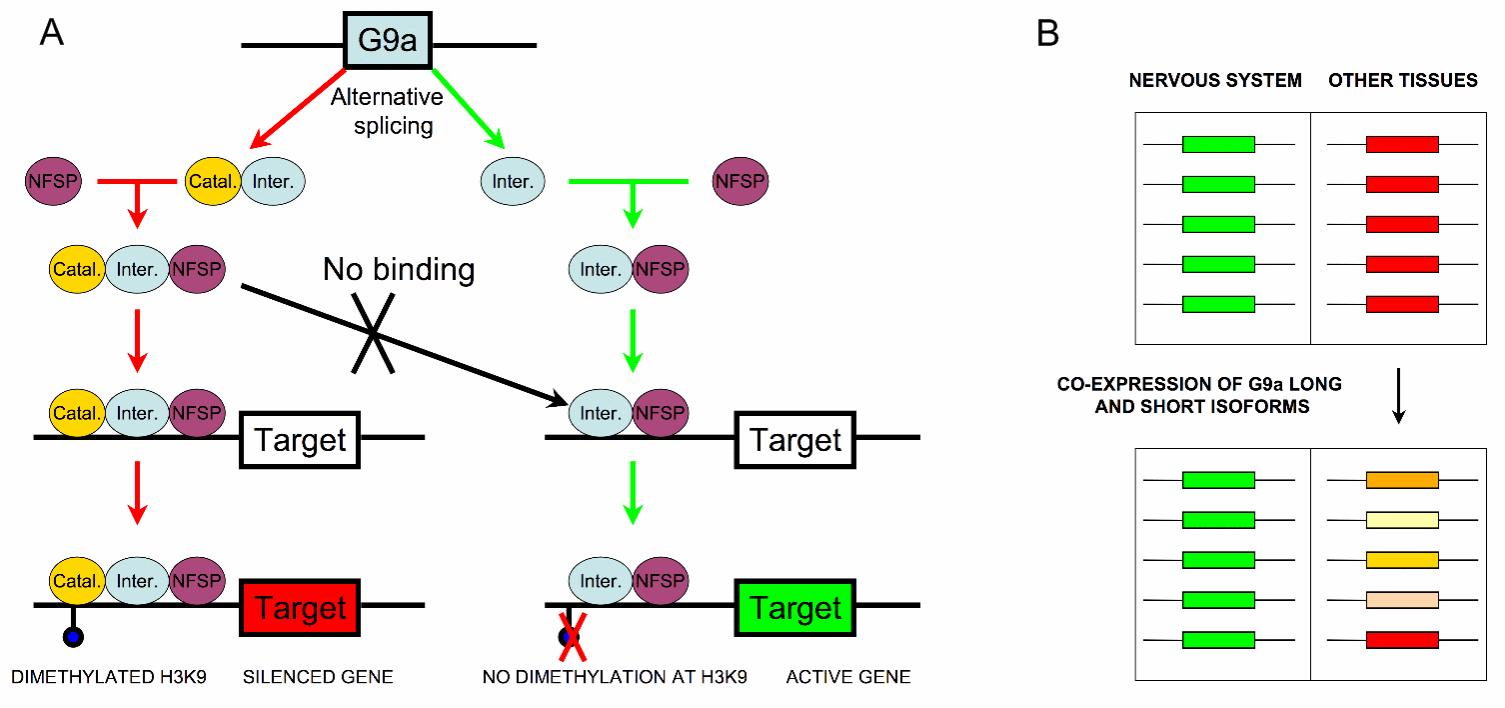
**Hypothetical mechanism of regulation by alternative splicing of histone dimethyltransferase G9a function**. (A) Experimental evidence indicates that histone dimethyltransferase G9a plays an important role in the silencing of neuronal genes in non-neuronal tissues [9]. In the proposed mechanism [9], shown here with red arrows, in non-neuronal tissues the transcription factor NFSP (shown in magenta) recruits the fully functional isoform of G9a (shown here with two domains: a binding domain in blue, and a catalytic domain in yellow) to a series of target genes that are subsequently silenced by G9a dimethylation of lysine-9 from histone H3. This mechanism may be inhibited/modulated by expression of the G9a short isoform (which only retains the NFSP transcription factor binding domain, Table 2), as shown here with green arrows. This isoform may behave as a dominant-negative inhibitor, as shown by the green arrows, blocking the access of the catalytically active isoform to the chromatin of the target gene. Absence of methylation marks in histone H3's lysine-9 would then result in an active gene. (B) The expression state of the target genes in both the nervous system (active, green colour) and in other tissues (silenced, red colour), as a result of the silencing, combined, action of NFSP and G9a. Co-expression of both the long and the short isoforms may result in the modification of the expression state of the target genes in non-neuronal tissues. These target genes may now show varying degrees of activity, as a result of the dominant-negative inhibitor role played by the short isoform (described in (A)).

Thus, our results show how alternative splicing may regulate the functional role of chromatin-modifying enzymes. This is a first step towards the goal of understanding the biological impact of alternative splicing on epigenetic gene expression regulation. This goal, which in general is very difficult to attain [17], becomes particularly hard in our case, as epigenetic regulators act both at gene-specific and whole-genome levels [2,40]. They are involved in relevant biological processes like development [5] or disease [6] and, in addition, they may also act on proteins other than histones. Nonetheless, our results clearly support the idea that alternative splicing is likely to have a substantial impact on the epigenetic regulation of large sets of genes, by regulating the activity of chromatin-modifying enzymes. One of the simplest mechanisms would be the co-expression of two alternative splice isoforms of one of these enzymes, a fully functional isoform and a dominant-negative inhibitor of the former, which may result in a reduced repression or activation of the set of genes controlled by this enzyme. To illustrate how this could happen, we can mention the case of G9a (EHMT2), a histone dimethyltransferase likely to play an important role in the repression of a large set of neuronal genes [9]. This repression, which can affect between 30 and 800 genes, is based on a chromatin-level mechanism [9] (Figure 6): (i) NFSP transcription factor would recruit histone dimethyltransferase G9a to the target genes; (ii) the latter would be silenced by G9a's dymethylation of histone tails at that location. It has been observed, that dominant-negative inhibition of G9a results in abrogation of this gene silencing [9]. In our case, we find that one of the G9a's isoforms has all the characteristics of a dominant-negative regulator (Table 2), as it has lost all its domains but the binding domain to NFSP transcription factor. We can speculate that this isoform could modulate the repression of this set of neuronal genes, in a similar way as G9a dominant-negative designed constructs [9] (Figure 6).

## Methods

### Dataset of epigenetic regulators

The list of chromatin-modifying enzymes was taken from five recent reviews on chromatin-modifying enzymes [2,3,19,41,42]. Note that DNA methyltransferases have not been considered. Subsequently we checked for the existence of alternative splicing for the corresponding genes in different databases: SwissProt [29], NCBI-Gene [43], Ensembl [44] and ASAP [45]. These databases have different annotation protocols, from manual annotation in SwissProt [29] to highly automatic procedures in Ensembl [44]. This allows increasing the coverage of our study. A discussion on possible error sources can be found at the end of the Materials and Methods section.

As shown in Table 5, the final dataset was constituted by 78 genes with alternative splicing, together with additional information on the species, protein name and function. Due to the different procedures followed in the different databases to obtain alternative splicing information we expect a complementary coverage of the alternative splicing patterns.

**Table 5:** List of genes showing alternative splicing

Gene Symbol	Species	Function	Protein name
CDY-1	H.s.	A	*chromodomain protein, Y-linked, 1*
GCN5L2	H.s.	A	*GCN5 general control of amino-acid synthesis 5-like 2 (yeast)*
HAT1	H.s.	A	*histone acetyltransferase 1*
HTATIP (TIP60)	H.s.	A	*HIV-1 Tat interacting protein*
MORF4L1	H.s.	A	*mortality factor 4 like 1*
MYST1	H.s.	A	*MYST histone acetyltransferase 1*
NCOA-1	H.s.	A	*Nuclear receptor coactivator 1*
TAF1 (TAF250)	H.s.	A	*TATA box binding protein (TBP)-associated factor, 250 kDa*
CARM1 (PRMT4)	H.s.	M	*coactivator-associated arginine methyltransferase 1*
DOT1L	H.s.	M	*DOT1-like, histone H3 methyltransferase (S. cerevisiae)*
EHMT2 (G9a)	H.s.	M	*euchromatic histone-lysine N-methyltransferase 2*
EZH2	H.s.	M	*enhancer of zeste homolog 2 (Drosophila)*
MLL	H.s.	M	*Myeloid/lymphoid or mixed-lineage leukemia*
PRMT1	H.s.	M	*protein arginine methyltransferase 1*
SETD8 (PR-SET7, SET8)	H.s.	M	*SET domain containing (lysine methyltransferase) 8*
SETDB1	H.s.	M	*SET domain, bifurcated 1*
SUV39H1	H.s.	M	*suppressor of variegation 3–9 homolog 1 (Drosophila)*
SUV39H2	H.s.	M	*suppressor of variegation 3–9 homolog 2 (Drosophila)*
ATM	H.s.	P	*ataxia telangiectasia mutated*
ATR	H.s.	P	*ataxia telangiectasia and Rad3 related*
AURKB	H.s.	P	*aurora kinase B*
MAP3K12 (DLK/ZIP)	H.s.	P	*Mitogen-activated protein kinase 12*
PRKDC (DNA-PK)	H.s.	P	*protein kinase, DNA-activated, catalytic polypeptide*
RPS6KA5 (MSK1)	H.s.	P	*ribosomal protein S6 kinase, 90kDa, polypeptide 5*
RPS6KA4 (MSK2)	H.s.	P	*ribosomal protein S6 kinase, 90kDa, polypeptide 4*
CHD-3	H.s.	R	*chromodomain helicase DNA binding protein 3*
CHD-4	H.s.	R	*chromodomain helicase DNA binding protein 4*
SMARCA1 (SNF2L)	H.s.	R	*SWI/SNF related, matrix associated, actin dependent regulator of chromatin, subfamily a, member 1*
SMARCA2 (BRM)	H.s.	R	*SWI/SNF related, matrix associated, actin dependent regulator of chromatin, subfamily a, member 2*
SMARCA4 (BRG1)	H.s.	R	*SWI/SNF related, matrix associated, actin dependent regulator of chromatin, subfamily a, member 4*
UBE2A	H.s.	U	*ubiquitin-conjugating enzyme E2A (RAD6 homolog)*
CKII	H.s.	P	*casein kinase 2, alpha 1 polypeptide*
EHMT1	H.s.	M	*Histone-lysine N-methyltransferase, H3 lysine-9 specific 5*
GSG2 (HASPIN)	H.s.	P	*Serine/threonine-protein kinase Haspin*
FBXL11 (JHDM1A)	H.s.	DM	*JmjC domain-containing histone demethylation protein 1A*
FBXL10 (JHDM1B)	H.s.	DM	*JmjC domain-containing histone demethylation protein 1B*
JMJD1B (JHDM2B)	H.s.	DM	*JmjC domain-containing histone demethylation protein 2B*
JMJD2B (JHDM3B)	H.s.	DM	*JmjC domain-containing histone demethylation protein 3B*
JMJD2C (JHDM3C)	H.s.	DM	*JmjC domain-containing histone demethylation protein 3C*
AOF2 (LSD1)	H.s.	DM	*Lysine-specific histone demethylase 1*
MLL2	H.s.	M	*Myeloid/lymphoid or mixed-lineage leukemia protein 2 (ALL1-related protein)*
MLL3	H.s.	M	*Myeloid/lymphoid or mixed-lineage leukemia protein 3 homolog*
WBP7 (MLL4)	H.s.	M	*WW domain-binding protein 7 (Myeloid/lymphoid or mixed-lineage leukemia protein 4) (Trithorax homolog 2)*
MLL5	H.s.	M	*myeloid/lymphoid or mixed-lineage leukemia 5 (trithorax homolog, Drosophila)*
NSD1	H.s.	M	*H3-K36-HMTase and H4-K20-HMTase*
PRMT5	H.s.	M	*Protein arginine N-methyltransferase 5*
PRDM2 (RIZ1)	H.s.	M	*PRDM2 (PR domain containing 2, with ZNF domain)*
RNF40	H.s.	U	*E3 ubiquitin-protein ligase BRE1B (RING finger protein 40)*
SETDB2	H.s.	M	*Histone-lysine N-methyltransferase SETDB2*
SIRT2	H.s.	DA	*NAD-dependent deacetylase sirtuin-2*
Gtf3c4	M.m.	A	*General transcription factor IIIC, polypeptide 4*
Htatip	M.m.	A	*HIV-1 tat interactive protein, homolog (human)*
Morf4l1	M.m.	A	*mortality factor 4 like 1*
Ncoa-1	M.m.	A	*Nuclear receptor coactivator 1*
Ehmt2	M.m.	M	*euchromatic histone lysine N-methyltransferase 2*
Ezh2	M.m.	M	*enhancer of zeste homolog 2 (Drosophila)*
Prmt1	M.m.	M	*protein arginine N-methyltransferase 1*
Carm1 (Prmt4)	M.m.	M	*protein arginine N-methyltransferase 4*
Setdb1	M.m.	M	*SET domain, bifurcated 1*
Suv39h1	M.m.	M	*suppressor of variegation 3–9 homolog 1 (Drosophila)*
Suv39h2	M.m.	M	*suppressor of variegation 3–9 homolog 2 (Drosophila)*
Stk4	M.m.	P	*serine/threonine kinase 4*
Myst2 (Hbo1)	M.m.	A	*Histone acetyltransferase MYST2*
Fbxl11 (Jhdm1a)	M.m.	DM	*JmjC domain-containing histone demethylation protein 1A*
Fbxl10 (Jhdm1b)	M.m.	DM	*JmjC domain-containing histone demethylation protein 1B*
Jmjd1a (Jhdm2a)	M.m.	DM	*JmjC domain-containing histone demethylation protein 2A*
Jmjd1b (Jhdm2b)	M.m.	DM	*JmjC domain-containing histone demethylation protein 2B*
Jmjd2a (Jhdm3a)	M.m.	DM	*JmjC domain-containing histone demethylation protein 3A*
Jmjd2b (Jhdm3b)	M.m.	DM	*JmjC domain-containing histone demethylation protein 3B*
Ring1A	M.m.	U	*E3 ubiquitin-protein ligase RING1*
Rnf20	M.m.	U	*E3 ubiquitin-protein ligase BRE1A*
Su(var)3–9	D.m.	M	*Suppressor of variegation 3–9*
trx	D.m.	M	*trithorax*
Taf1	D.m.	P	*TBP-associated factor 1*
brm	D.m.	R	*Brahma*
cbp-1	C.e.	A	*Bromodomain*
fbxl10 (jhdm1b)	X.l.	DM	*JmjC domain-containing histone demethylation protein 1B*
mez2	Z.m.	M	*Polycomb protein EZ2*

In the "Species" column H.s., M.m., C.e., O.s., X.l. and Z.m. mean *Homo sapiens*, *Mus musculus*, *Caenorhabditis elegans*, *Oryza sativa, Xenopus laevis *and *Zea mays*, respectively. In the column "Function" A, DA, DM, M, P, U and R mean Acetylation, deacetylation, demethylation, methylation, phosphorylation, ubiquitination and chromatin remodelling, respectively.

In general, the gene names used follow the international standards set for each species. Standard gene names were obtained: for human from the Human Gene Nomenclature Database [46]; for mouse from the Mouse Genome Database (MGD) [47]; for *D.melanogaster *from the FlyBase [48], version FB2006_01; for *C.elegans *from the WormBase [49], release WS166; for *Z.mays *from MaizeGDB [50].

The detailed exon structure of the isoforms studied in this work is provided in an additional file [see Additional file ExonStructure.xls].

### Possible error sources

As explained in the previous section, alternative splicing data are obtained from different databases and come from different sources -e.g. literature, processing of ESTs- therefore they will have a different error attached to them. Unfortunately, it is not possible to provide a reliability measure for each observation, but we can discuss the reliability of the general trends observed and how the possible sources of error affect the main conclusions of our work.

First, we observe that the overall trends we find in our dataset coincide with those previously observed by other authors that have studied alternative splicing in more general sets of genes. In particular, the fact that insertions/deletions of domain size prevail in our dataset is in agreement with previous observations [39]. Also the corresponding mechanisms for function modulation -dominant-negative inhibition, amount regulation- have been proposed and observed for other genes [17], although the biological context and expected impact are obviously different. Some of the very short isoforms we have obtained can be artifactual but they may also constitute a possible regulatory mechanism [51]. In fact very short isoforms have been described for the genes in our study, e.g. for MLL [52].

At a more detailed level, in the case of data from ASAP [45], the authors provide an error estimate of less than 2 % [53]. To decrease it more, we discarded all the ASAP isoforms for a given gene, when none of them coincided with the longest isoform provided by another database. For the remaining databases the error estimates will vary, even within the database. For example, in the case of SwissProt [29], protein records are manually annotated, but the evidence supporting a given isoform may vary from one gene to another. Nonetheless, SwissProt [29] has been utilised in many bioinformatics studies on alternative splicing due to the high quality of the data [39,54-59]. In the case of Ensembl [44], the predictive nature of the annotations suggests that there may be a certain amount of false positives. The latter may be more frequent in the case of very short isoforms, although it has to be mentioned that these isoforms are usually supported by a substantial amount of evidence from EST data and other databases.

For all these reasons, we believe that the overall conclusions of this work will not be substantially affected by possible errors in the data.

### Domain annotation

The domain structure of the different isoforms was obtained utilising CD-Search [60]. This program identifies the functional domains present in a protein sequence. We focused our analysis on the Pfam [61] and Smart [62] domain definitions. COG (Tatusov et al., 2001) definitions were not available for all the species and for this reason they were not utilised (no significant differences were observed when utilised in this analysis). Because in some cases domain boundaries for the same domain would change slightly from one database to another, we combined the two definitions in a consensus domain definition, as follows: the location of the N-terminal domain was taken to be the minimum of the Pfam [61] and Smart [62] values; for the C-terminal end, instead of the minimum, we took the maximum of the Pfam [61] and Smart [62] values. For example, if a given domain occupies positions 3–75 and 8–82 according to the Pfam and Smart definitions, respectively, in our consensus definition it will go from position 3 to position 82.

We eliminated from the domain mapping all the domains with functional annotations of no, or unclear, meaning within the context of this work, that is: microbial domains, like viral capsid domains, and Pfam B domains [61]. In Table 6 we provide a list of the domains affected by alternative splicing mentioned in this work.

**Table 6:** List of domains affected by alternative splicing in chromatin-modifying enzymes

Domain name	Function	Enzyme name
AMINO_OXIDASE	Catalytic	AOF2
ANK	Protein-Protein Interaction	EHMT1, EHMT2
BRK	Unknown	SMARCA2, SMARCA4
BROMO	Interaction (Acetylated Lysines)	SMARCA2, SMARCA4, MLL
CHROMO	Interaction (Methylated Lysines)	SUV39H1, SUV39H2, Suv39h2, Su(var)3–9, MYST-1, MORF4L1, Morf4l1, Htatip
DEXHC	Catalytic	SMARCA1, SMARCA2, SMARCA4
FAT	Interaction/Modulate catalysis	ATM
FATC	Interaction/Modulate catalysis	ATM
FYRC	Probably not-catalytic	MLL, WBP7
FYRN	Probably not-catalytic	MLL, WBP7
HELICASE_C		SMARCA2
HSA	Probably DNA binding	SMARCA2, SMARCA4
JMJC	Catalytic	FBXL11, fbxl10 (from Mus musculus and Xenopus laevis), jmjd1b
LRR_RI	Interaction	FBXL10
MBD	DNA binding	SETDB1, Setdb1
MOZ_SAS	Catalytic	Htatip
MRG	Interaction	MORF4L1
PCAF_N	Interaction with CBP	GCN5L2
PHD	Intra- and Intermolecular interactions	MLL, MLL2, MLL3, JMJD2B, Jmjd2a, WBP7
PI3_PI4_KINASE	Catalytic	PRKDC, ATM
PKINASE	Catalytic	AURKB, GSG2, RPS6KA5, stk4
PRESET	Interaction-Catalysis	SUV39H1, SUV39H2, Suv39h2, Su(var)3–9, SETDB1, Setdb1, EHMT1, EHMT2
PWWP	Unknown	NSD1
RING	Interaction	MLL2, RNF40
SET	Catalytic	PRDM2, SUV39H1, SUV39H2, suv39h2, Su(var)3–9,SETDB1, Setdb1, mez2, MLL, WBP7, EHMT1, EHMT2, EZH2
SKB1	Catalytic	CARM1
UBCC	Whole protein	UBE2A
TUDOR	Interaction	Jmjd2a, Jmjd2b, JMJD2B, SETDB1, Setdb1
ZF_C3HC4	Interaction	RNF40
ZF_CXXC	Interaction	Fbxl10, Fbxl11, MLL, WBP7,
ZNF_TAZ	Interaction	cbp-1

### Classification of the alternative splicing events

Our study focused on those alternative splicing events that affect any of the known domains, as it is easier to infer their functional impact [17]. In general, epigenetic regulators are multidomain proteins that have both catalytic and interaction domains. Because the functional role of a given isoform will depend on which of these domains has been affected by alternative splicing, we grouped the observed isoforms according to the biochemical nature of the affected domain(s): (i) alternative splicing affects the catalytic domains; (ii) alternative splicing affects the protein interaction domains; and (iii) alternative splicing affects results in drastic sequence reductions. An alternative splicing event belongs to the first class when the corresponding sequence change mainly affects the catalytic domains, but the resulting isoform retains at least one of its binding domains (i.e. keeps its binding ability). Alternative splicing events are classified in the second group when the sequence change mainly affects the interaction domains, but not the catalytic unit. Finally, alternative splicing events belong to the third class when both the catalytic and the binding domains are affected by the sequence change. Four proteins were not included in this classification, ubiquitin-conjugating enzyme E2A (UBE2A, 154 aas), casein kinase 2, alpha 1 polypeptide (CKII, 391 aas), NAD-dependent deacetylase sirtuin-2 (SirT2, 389 aas) and aurora kinase B (AURKB, 344 aas) because they only have a single domain which plays both a catalytic and a binding role and therefore large alternative splicing sequence changes are very likely to affect both functions simultaneously.

### Structure analysis

Direct structural information was not available for none of the proteins considered in this work. However, in some cases the changes produced by alternative splicing embraced a part of the sequence for which structural information was available from a homolog. In these cases, this part was modelled utilising the well known, standard, modelling package MODELLER [63], and using the structure of the homolog as a template. The latter was obtained from the PDB database [64]. A list of cases, together with the domains involved, the homologs utilised, and the sequence identities between the latter and our proteins, is shown in Table 7.

**Table 7:** Templates utilised for comparative modelling

Protein name	Size Ref.	Species	Domain name	PDB code	% Seq. Id.
cbp-1	2056	C.e.	ZNF_TAZ	1L8C	75
SMARCA1 (SNF2L)	1054	H.s.	DEXHC	1Z6A	38
SMARCA2 (BRM)	1590	H.s.	BROMO	1N72	26
SUV39H2	410	H.s.	CHROMO	1KNA	47
SUV39H2	410	H.s.	SET	1MVH	39
UBE2A	154	H.s.	UBCC	1JAS	95
PRKDC (DNA-PK)	4127	H.s.	PI3-PI4 KINASE	1E8Y	29

In the "Species" column H.s. and C.e., mean *Homo sapiens *and *Caenorhabditis elegans*, respectively. The size of the whole protein is given in amino acid number. % Seq.Id. is the percentage of sequence identity between the target and the template sequences. The PDB code is the code of the template structure utilised for the comparative modelling in the PDB database [64].

Structural models are utilised throughout the article to illustrate the location of alternative splicing changes and to help understand/infer their functional impact. The conclusions that can be drawn from the use of these models are limited by the following facts: (i) in general, epigenetic regulators are multidomain proteins, while the structures correspond to only one of these domains; (ii) the structural changes resulting from certain sequence changes may be difficult to predict. It is clear that the structural analysis would benefit from taking into account the structure of the whole protein, but this information is not yet available for the proteins in our dataset or for their homologs, neither close nor remote. This would be a serious problem if our aim were to predict with high accuracy the structural/functional changes resulting from alternative splicing. However, our goal is more coarse-grained, as what we want to see is whether alternative splicing changes result in the presence or absence of the biochemical function associated to a given domain. When the sequence change affects the whole domain, by far the most frequent situation, it is reasonable to assume that the resulting protein has lost this activity and that it may function as a regulator (e.g. a dominant-negative inhibitor) of the full-length isoform, something that has been experimentally confirmed in the case of transcription factors [17], among others.

If the sequence change does not reach the domain size the situation is more complex, because it is more difficult to decide whether it will result in complete function loss, modulation of an original function or creation of a new function. Without further structural data we cannot provide a definite answer for none of our cases. However, in some instances the nature of the sequence change is not compatible with preservation, or smooth modulation, of the domain's function. This happens when the domain is small and the sequence change is large, or it affects the protein core or any important secondary structure element. In these cases we have proposed that the most likely effect of alternative splicing is that of a regulator of the fully functional isoforms, something that has been already observed in the case of the epigenetic regulator SMARCA1 [22].

Finally, we cannot reject the possibility that some of the regions affected by alternative splicing may be intrinsically disordered, as has been recently proposed [59]. However, if the sequence stretch affected by alternative splicing encompasses a whole protein domain the functional interpretation will remain the same, as it is independent of whether the domain in question is structured or disordered. If the affected stretch is of sub-domain size, the situation could be different if we knew that the domain involved is disordered. However, this is unlikely as the domains affected by alternative splicing discussed here are homologues, sometimes very close, of domains with known three-dimensional structure (Table 7).

## Abbreviations

aas: amino acids.

## Authors' contributions

SL obtained the set of manually curated data, annotated them with the alternative splicing and protein domain information. NB contributed to design the study and to its testing. MM-B and XdC conceived the study, designed most of the testing and wrote the article. All authors read and approved the final manuscript.

## Supplementary Material

Additional file 1 Exon structure of the isoforms studied. The file provides a description of the exon structure of the isoforms analysed in the present article (Table 2). Most of the data were obtained after querying the ENSEMBL [44] and NCBI Gene databases [43]. Part of the data were also obtained after aligning the target isoform with the genome of the corresponding species or using the SEDB package [66]. Finally, in four cases (GSG2, Jmjdb1, fbxl10 and mez2, from human, mouse, frog and maize, respectively) no information could be retrieved. The structure of the file is the following: the first column corresponds to the name of the genes; the second column corresponds to the isoform size; the third column corresponds to the organism; and the following columns correspond to the exons constituting the isoform. Each gene is preceded by a line with these fields and the order of each exon within the gene (exons with no order number correspond to parts of the isoform sequence for which the exon could not be identified). For each gene the data given in the first line correspond to the longest, full-length, isoform; data in the following lines correspond to the remaining isoforms. The numbers within each exon cell correspond to its size in amino acids. A colour code was used to distinguish constitutive exons (red), alternative initiation sites (yellow), intron retentions (green), and sequence stretches with no exon(s) assigned (lilac). Click here for file
